# Privacy and uniqueness of neighborhoods in social networks

**DOI:** 10.1038/s41598-021-94283-5

**Published:** 2021-10-11

**Authors:** Daniele Romanini, Sune Lehmann, Mikko Kivelä

**Affiliations:** 1 Department of Computer Science, Aalto University, 02150 Espoo, Finland; 2 DTU Compute, Technical University of Denmark, 2800 Lyngby, Denmark

**Keywords:** Applied mathematics, Computational science, Computer science

## Abstract

The ability to share social network data at the level of individual connections is beneficial to science: not only for reproducing results, but also for researchers who may wish to use it for purposes not foreseen by the data releaser. Sharing such data, however, can lead to serious privacy issues, because individuals could be re-identified, not only based on possible nodes’ attributes, but also from the structure of the network around them. The risk associated with re-identification can be measured and it is more serious in some networks than in others. While various optimization algorithms have been proposed to anonymize networks, there is still only a limited theoretical understanding of which network features are important for the privacy problem. Using network models and real data, we show that the average degree of networks is a crucial parameter for the severity of re-identification risk from nodes’ neighborhoods. Dense networks are more at risk, and, apart from a small band of average degree values, either almost all nodes are uniquely re-identifiable or they are all safe. Our results allow researchers to assess the privacy risk based on a small number of network statistics which are available even before the data is collected. As a rule-of-thumb, the privacy risks are high if the average degree is above 10. Guided by these results, we explore sampling of edges as a strategy to mitigate the re-identification risk of nodes. This approach can be implemented during the data collection phase, and its effect on various network measures can be estimated and corrected using sampling theory. The new understanding of the uniqueness of neighborhoods in networks presented in this work can support the development of privacy-aware ways of designing network data collection procedures, anonymization methods, and sharing network data.

## Introduction

Much of the complexity of social systems, ranging from individual social groups to whole societies, is encoded in the structure of the interactions between individuals. These *social networks* are a focal point in quantitative research aiming to explain how social systems work from the microscopic level of individuals to the macroscopic level with millions of people. This area of research spans studies from the beginning of the previous century^1^ to more recent explosion of work on automatically collected digital traces of up to millions of individuals. The topics of this research are numerous and are related to many of the burning issues in the world such as opinion polarisation^2–4^, disease spreading^5–7^, and social segregation^8–10^, to name a few. What is common to this wide range of research is that it is all based on data describing the structure of social networks, represented simply through nodes (individuals) and edges (their connections).

In order for the scientific community working on social networks and related problems to function efficiently, it is imperative that researchers can share social network data. First, only sharing network data allows scientists to reproduce already presented results and validate (or rebut) them. This process is the basis of the scientific method, and essential in order to avoid a crisis in reproducing scientific results which have already been reported in multiple fields^11–13^. Second, social network data can be (and is routinely) reused to answer questions that were not conceived by the original research group releasing the data. In some cases, the social network data itself could turn out to be more important for the scientific progress than the answer related to the original research question that was answered in the article releasing the data.

Social network data can contain sensitive information which can make it impossible to share such data due to privacy concerns. For example, the structure of the network itself, i.e. who connects to whom, can be sensitive — or the individuals could have sensitive attributes attached to them. As networks contain complex multidimensional information compared to, for instance, tabular data, they result in a complex problem of how to share data in a privacy preserving way.

Many of the data anonymization techniques developed over the years are simply unsuitable for social network data. A specialised set of network-based techniques have been developed to address this problem. Within these there are a number of different scenarios (threat models) of how exactly a malicious party might be able to deanonymize the network, and even greater number of methods to anonymize network data (see “Privacy in social networks”). The focus in the literature has been on developing methods that minimize the changes to the networks — or particular network statistics — while making them less vulnerable to a particular deanonymization attack.

In this paper we study the network anonymization problem by analysing which factors that tend to make networks vulnerable in the first place. We do this by studying different network models, varying their parameters and measuring their vulnerability in various configurations. A better understanding of the causes of vulnerability can both guide to a better design of solutions to address each vulnerability and, more importantly, lead to a more accurate risk assessment. Based on our findings, we explore a simple anonymization strategy based on random edge sampling to choose which edges to keep in the original network (or equivalently, random edge removal). Here, the idea is that changes to the networks can be substantial as long as they are statistically tractable such that they can be corrected for further analysis. This is in contrast with optimization-based method, which may result in arbitrary changes to the networks structure. The effects of these methods are not easily reversible, making it hard to for the user of the anonymized data to reconstruct original statistics and estimate the error in them.

We focus on a single well-studied threat model known as the neighborhood attack^14^. In this scenario the attacker has knowledge of the structure of the network neighborhood of a victim node, which is represented by the number of a person’s friends and the connections between them. Based on this information, the attacker tries to re-identify the victim in the anonymized network. If a node is re-identified in this way, the attacker could get access to private information potentially attached to the node, or the wider position of the node in the network. Furthermore, the attacker could also re-identify the target’s connections (e.g. friends or relatives). The latter case could be possible also if an individual who knows the structure of their own neighborhood is able to recognize himself in a certain dataset. In this case, then they would potentially be able to re-identify their friends and their connections. The links themselves, even without explicit labels, could be already a private information (as they represent, for example, the people we communicate or spend time with), and thus important to protect.

The remainder of this paper is organized as follows. In “Privacy in social networks”, we give an overview of the already existing works on privacy in social networks, in particular on neighborhood attack. In “Measuring privacy risks with uniqueness of neighborhoods”, we formally introduce the threat model we use and the related network diagnostics. We present our main findings in “Uniqueness of neighborhoods in random network models” and “Uniqueness of neighborhoods in empirical networks”, showing how network models can also be a good proxy for real-world networks with respect to uniqueness of neighborhoods. Finally, in “Mitigating neighborhood attacks”, we show how our findings can be applied in practice to lower the re-identification risk in networks.

## Privacy in social networks

Privacy in data sharing is a growing area of research. With increasing amount of personal data and data analysis techniques, the risk for users’ privacy has dramatically increased. Existing privacy-preserving and anonymization methods often rely on different definitions of privacy that have been mainly developed for tabular data, even though there has been some efforts to extend those definitions to networks.

Some of the most popular existing definitions of privacy are: *naïve anonymization* or *pseudonymization*, which consists in dropping the entities’ labels, and replacing them with random labels; *random perturbation/noise injection*^15^; *k-anonymity*^16^, where the dataset is modified such that each entry is indistinguishable from at least other $k-1$ entries (or, in other words, each equivalence class contains at least *k* values), reducing the re-identification attack success probability to $\frac{1}{k}$; $\ell$*-diversity* and *t-closeness*, which are group-based anonymization techniques for labelled data, developed to strengthen the definition of privacy given by *k-anonymity*; *Differential privacy*^17,18^, a mathematical definition used to develop algorithms to query data, ensuring the privacy of the response, or, more recently, to learn generative models for private data sharing^19^. Some differential privacy methods have been developed to perform specific network analysis tasks^20–22^, aiming to protect only some information, such as the ones related to nodes (node differential privacy^23^) or edges (edge differential privacy).

In networks, in addition to node-attributes, there is a further threat due to the network structure itself. That is, a node or a link can be identifiable by its “location” in the network. In fact, without this consideration, one could think that a naïve anonymization approach could be enough for sharing network data. To account for these structural threats, the private network components, such as nodes or links, need to be structurally indistinguishable in the network as a whole. To address these type of general structural attacks, concepts such as *k-automorphism*^24^ and *k-isomorphism*^25^ have been developed. These assess the privacy risks arising from an the attacker with knowledge of the whole graph or any part of it.

An alternative to anonymizing the whole network structure is to compute an array of network statistics, anonymize the array, and either share the statistics or uniformly sample a network with the set of anonymized statistics. One such approach is to share the block matrix of a stochastic block model^26^. Further, some papers have proposed sharing the *dK*-series^27^ or a hierarchical random graph^28^ under differential privacy. Differential privacy is certainly the state-of-the-art in data privacy research, and those studies proposed promising directions for private data-sharing. However, these statistics-based methods have the serious drawback that they only retain the particular statistics that are measured; or in the case of models, structure that is encoded in each model. For example, the *dK*-series is often used to retain degree correlations ($dK-2$-series) (and possibly triangle counts)^29^, but disregard any mesoscopic structures, such as a particular community structure. Further, depending on the statistics, it can be difficult to develop algorithms which are guaranteed to sample uniformly from networks with specific set of statistics^30^.

An attacker does not need to have a knowledge of the full network, but some local structural features can make a node unique and thus re-identifiable in a network. Typical local features like this include the number of connections (degree) or the structure of its neighborhood. This notion has been formalized as *neighborhood attack*, which consists in the re-identification of a node based on its neighborhood^14^. This work initially focused on the 1-hop neighborhood of a node, which is composed by its immediate neighbors only, in unweighted, undirected networks. Related concepts such as *k-degree anonymity*^31^ and *k-neighborhood anonymity*^14^, were developed to asses the risk of attacks such as the neighborhood attack.

Several algorithms have been developed for making networks safe from neighborhood attacks, while changing the network a minimal amount. Finding an optimal solution (e.g. adding the minimum amount of edges) to reach the anonymity is a NP-hard problem^32^, and in general solving it needs to be done via heuristic algorithms. For example, one heuristic anonymizes pairs of neighborhoods (starting with the ones with the highest amount of vertices) making them isomorphic if they are not already, by adding edges^14^. This approach has been developed further by representing the neighborhood components with adjacency matrices, making the procedure extensible to more than 1-hop neighborhood^33^. Focusing on the number of edges that are changed during the anonymization is not the only possible approach, but, depending on the application, one can also focus on specific network metrics. For example, distances between pairs of nodes have been used as such metric^34^.

Several network datasets come with some additional information on the nodes or the edges in addition to the network structure. In fact, studies have shown that attributes and meta-data can be crucial to identify individuals in some datasets, such as metadata associated to social media usage, credit card transactions, and visited locations^35–37^. Clearly, the uniqueness of subjects in a dataset varies with the amount of detailed information the data expose. An illustrative example of this is a study exploring the uniqueness in function of the temporal and spatial resolution of mobility traces of people^37^. In fact, there is a parallel between this study on resolution and our work: we can think of the location visited of as one type of nodes of a bi-partite graph, which are connected to the people, represented by the other type of nodes of the same graph. If we increase the spatial resolution, the number of possible nodes will increase, and so the average degree of the people’s nodes.

Neighborhood anonymization methods have been developed for anonymizing labelled neighborhoods^38,39^, in an attack where the attacker has also a background knowledge of the labels of the nodes part of the target’s neighborhood, besides of its structure. Additional information in links can be considered by treating the problem of neighborhood anonymization in weighted unlabelled networks, and anonymizing the network by edge additions and weight modifications^40^ .

Network anonymization algorithms which modify networks by addition or removal of nodes and links aim to minimize such changes. In general, there is always a balance between utility and anonymity of the resulting network. Depending on the data, with high privacy requirements the amount of changes to the network can be very significant. Further, while the anonymizer knows where exactly these changes are and can evaluate their impact to specific network metrics, the end user of the anonymized data must assume that the changes could be in arbitrary places in the network. This makes it difficult for users to evaluate the errors or make the error estimates very high even for moderate amount of changes. For example, the impact to performance on classification methods such as Graph Neural Networks has been show to be significant when the worst-case places for node or edge additions are assumed^41–43^.

The literature on network privacy and anonymity has focused on developing algorithms that ensure various notions of anonymity while minimizing particular types of changes to networks. Much less work has focused on understanding the factors that cause networks to be anonymous in the first place. For example, given a threat model, such as neighborhood attack, it would be useful to know how difficult the anonymization problem is, and “how far” the original network is from having zero unique neighborhoods. In fact, if the number of unique neighborhoods is very high, it might be not worth-while to anonymize and share the network at all, as too many modifications would be required, significantly lowering the data utility. Ideally we would also like to have an (approximate) understanding of this difficulty without seeing the full network data.

Moreover, the local network topology plays an important role both from an analysis perspective (e.g. to measure how dense communities are), and from a privacy perspective. In fact, it has been shown that an attacker with an auxiliary graph (e.g. from a public source) could potentially re-identify nodes based on the structure of the graph itself^44^. For this reason, we believe that studying the structural properties that make networks vulnerable to unique re-identification of nodes are important, even disregarding the labels that would possibly be provided.

From the existing literature we understand theoretically how the knowledge of nodes degree, degrees of neighbors, degrees of neighbors’ neighbors and so on, can be used to deanonymize nodes in networks produced by the Erdős–Rényi model at the limit of infinite size^26^. In these large networks, the degree is not enough to uniquely identify a node, but, for successive higher-order degrees, uniqueness depends on the network density. Further, individual instances of power-law networks and lattices have been analysed with the same approach^26^. Our current work is similar in nature to this previous study, but we assume the more common neighborhood attack scenario where the attacker has knowledge about the entire target’s neighborhood structure. We further take into account finite size Erdős–Rényi (ER) networks, analyzing simultaneously both size and density, but focusing on sparse networks, as real social networks are typically sparse. As sparse ER networks are different from real social networks, since almost completely void of local structure, we also analyze the Watts–Strogatz, the Barabási–Albert and the Random Geometric Graph models which are minimal models containing such structure.

## Measuring privacy risks with uniqueness of neighborhoods

We want to study the uniqueness of neighborhoods in networks, to understand the factors that influence uniqueness in different settings and, at the same time, shed light on which network properties are relevant to quantify the privacy risk. The neighborhood of a node *v* consists of both the nodes which are adjacent to it and the links between them. Formally, we define the neighborhood, ${\mathscr {N}} (v)$, to be the induced subgraph of its neighbors. That is, given a graph $G=(V,E)$, ${\mathscr {N}} (v) = (V(v),E(v))$, where $V(v)=\{(u \in V | (v,u) \in E\}$ and $E(v) = \{(u,w) \in E | u,w \in V(v)\}$. A neighborhood of a node is unique if there are no other node neighborhoods in the network with the same graph structure (disregarding the node labels). Formally this means that ${\mathscr {N}} (v)$ is unique if there is no other node $u \in V$ for which the neighborhoods are graph isomorphic, ${\mathscr {N}} (v) \cong {\mathscr {N}} (u)$ (where ‘$\cong$' indicates graph isomorphism between the two graphs ${\mathscr {N}} (v)$ and ${\mathscr {N}} (u)$).

The uniqueness of a neighborhood guarantees that an attacker equipped with the knowledge of the neighborhood of a node can identify with absolute certainty the given node. However, a stronger notion of privacy can be achieved if there are multiple neighborhoods with exactly the same structure.

We define the *occurrence frequency* $O_{{\mathscr {N}} (v)}$ as the number of neighborhoods in *G* that are isomorphic to ${\mathscr {N}}(v)$:

1 $$\begin{aligned} O_{{\mathscr {N}} (v)} = \sum _{u \in V}{\delta ({\mathscr {N}} (v) \cong {\mathscr {N}} (u) )} \,, \end{aligned}$$

where

2 $$\begin{aligned} \delta ({\mathscr {N}} (v) \cong {\mathscr {N}} (v')) = {\left\{ \begin{array}{ll} 1, &{} \,if\,{\mathscr {N}} (v) \cong {\mathscr {N}} (v')\\ 0, &{} \,otherwise \end{array}\right. } \,. \end{aligned}$$

In order to quantify the overall privacy of a network, we define the *uniqueness of neighborhoods* $U_{{\mathscr {N}}}$ (or, simply, *uniqueness*, in our case) as the fraction of nodes with unique neighborhood structure in a network:

3 $$\begin{aligned} U_{{\mathscr {N}}} = \sum _{v \in V}{\frac{\delta (O_{{\mathscr {N}} (v)} = 1)}{|V|}} \,, \end{aligned}$$

where:

4 $$\begin{aligned} \delta (O_{{\mathscr {N}} (v)} = 1) = {\left\{ \begin{array}{ll} 1, &{} \,if\,O_{{\mathscr {N}} (v)} = 1\\ 0, &{} \,otherwise \end{array}\right. } \,. \end{aligned}$$

If the value of uniqueness is equal to one (maximum uniqueness), it means that there are only unique neighborhoods in the network, thus no neighborhood is isomorphic to any other. Conversely, if $U_{{\mathscr {N}}}=0$ (minimum uniqueness), every neighborhood occurs at least two times in *G*, and if $U_{{\mathscr {N}}}=0.5$, half of the neighborhoods occur just one time in *G*.

Essentially, the uniqueness of neighborhoods is equivalent to the number of neighborhoods in the graph that do not satisfy *k-anonymity* with $k \ge 2$. That is, it corresponds to the number of graph isomorphism classes to which one and only one neighborhood belongs.

A node could be uniquely identifiable not only by its neighborhood, but also by its degree. We define the *degree uniqueness* $U_{k}$ of a network *G*, as the fraction of nodes in *G* that have unique degree. Equations (1), (2), (3) and (4) can still be applied to the this type of uniqueness, by substituting the neighborhood ${\mathscr {N}}(v)$ with a function *k*(*v*) which returns the degree of node *v*.

Clearly, $U_{{\mathscr {N}}} \ge U_{k}$, because if a node has a unique degree, it also has a unique neighborhood. The two notions of uniqueness can differ from each other when there is at least one edge between the neighbors of the central node. Each edge in a neighborhood corresponds to a triangle where the central node is participating, and, in “Uniqueness of neighborhoods in random network models”, we discuss how the presence of triangles affects the value of $U_{{\mathscr {N}}}\,$ and its difference with $U_{k}$. For this reason, we introduce the notation for the fraction of nodes with degree *k* having at least one triangle in their neighborhood as $p_{k}$, and the expected fraction of neighborhoods with at least one triangle as ${\mathscr {N}}_{\bigtriangleup }$.

In the following, we study structural privacy, and, in particular, neighborhood anonymity, in unlabelled, unweighted, undirected networks with no self-loops. We study the case of 1-hop neighborhoods only, but our methodology can be adapted to higher order neighborhoods or to other structures that can uniquely characterize a node, for instance the degree, or labelled neighborhoods.

## Uniqueness of neighborhoods in random network models

In order to understand how networks behave in terms of uniqueness of neighborhoods, and thus vulnerability to neighborhood attacks and difficulty of the anonymization problem, we study four different network models. We compute the expected uniqueness values of networks produced by these models. The models we study are Erdős–Rényi (ER)^45^, the Watts–Strogatz (WS)^46^, the Barabási–Albert (BA)^47^ and a Random Geometric Graph (RGG)^48^ model. We choose those models because we want to represent different levels and aspects of randomness and local structure. All models are specified using two parameters; here we choose to represent them with the size and average degree. We note that there are of course several other models for social networks (and other networks), which we have left out, and which can produce a rich set of structures such as fat-tailed degree distributions, homophily, community structure, core-periphery structure^8,9,49,50^.

The ER model generates networks with nodes that are randomly connected by an edge with probability *p*. In large and sparse ER networks almost all neighborhoods are empty, and, as we explain in “Degree and neighborhood uniqueness in Erdős–Rényi networks”, there is a parameter range where ER networks are unlikely to be useful proxies for estimating uniqueness for real-world networks, since the latter typically have important local structure. We could pursue a similar line of reasoning for the BA model, which also produces networks with empty neighborhoods in the large and sparse regime. However, compared to ER (or WS) model, BA has a power-law degree distribution ($P(k) \sim k^{-3}$), which is more similar to many real-world networks^47^) and makes nodes heterogeneous in terms of degree. In the BA model, a network is generated by adding one node at a time and connecting it with already existing nodes, with a preferential attachment model (i.e. a link to an existing node is created with a probability proportional to the number of edges that are already connected to that node).

In contrast to ER and BA, the WS and the RGG can be used to generate networks with realistic neighborhood densities (i.e., clustering coefficient values). The WS model has a parameter $\beta$ representing the probability of rewiring each edge from a regular lattice structure (if $\beta = 1$, the generated graph is a random graph similar to a ER graph). The RGG (in its soft version, which we use) is constructed by randomly placing *n* nodes in an Euclidean space uniformly. If two nodes are within a given radius *r*, they are connected with a specified probability (in our case, an exponential distribution). The expected average degree of RGG is roughly $\langle k \rangle \approx \pi (n-1) r^2$. The WS and RGG models are not as easily amenable to derivations of explicit equations for uniqueness as the ER model, and instead, in “Uniqueness maps”, we show the uniqueness values for suitable parts of the parameter space of all of the models.

### Degree and neighborhood uniqueness in Erdős–Rényi networks

**Figure 1:**
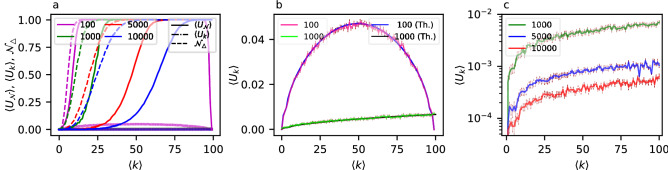
Neighborhood uniqueness, degree uniqueness and non-empty neighborhoods in ER networks with varying size and average degree. (**a**) The expected uniqueness of neighborhoods $\langle U_{{\mathscr {N}}} \rangle$ (continuous lines), degree uniqueness $\langle U_{k} \rangle$ (semi-continuous lines, on the bottom of the panel) and number of neighborhoods ${\mathscr {N}}_\Delta$ with at least one triangle (or, non-empty neighborhoods, in dashed lines) in ER networks of size 100, 1000, 5000 and 10,000 (computed as the mean of 10 independent network realizations), for values of average degree from 0 to 100. The degree uniqueness values are very close to 0 and for network sizes from 1000 to 10,000 they are below 0.02 and covered by each other. See panels (**b,c**) for a zoom of those curves. (**b**) Expected degree uniqueness for networks of size 100 and 1000 both from simulations, and the theoretical line, computed with Eq. (5). The means and errors (computed as standard error of the mean) are computed over 400 independently simulated networks for the networks with 100 nodes, and 20 independently simulated networks for the ones with 1000 nodes. This is because those are bigger systems, thus self-averaging, i.e., the variance between samples of the larger networks are smaller. (*c*) Expected degree uniqueness $\langle U_{k} \rangle$ (in a log scale) for networks of size 1000, 5000 and 10,000, for a range of average degree $\langle k \rangle$ from 0 to 100. The error bar represents the standard error of the mean. The means and errors are computed over 20 independently simulated networks. In this panel, the vertical axis representing the expected degree uniqueness is in log scale.

We start our investigation by analyzing the Erdős–Rényi (ER) model. The ER is arguably the model, with adjustable size and average degree, that has the minimum number of assumptions^51^, and it has been analysed previously at the limit of infinite network size under a different attack model^26^. As shown in the examples of uniqueness as a function of degree for networks of sizes 100, 1000, 5000 and 10,000 in Fig. 1a, uniqueness in the ER networks is sensitive to both size and average degree. Simply analysing the limiting behavior in the size is not sufficient.

When computing the neighborhood uniqueness in ER networks as a function of average degree, the uniqueness is at first a monotonically increasing function until it hits the maximum value ($U_{{\mathscr {N}}}=1$). On the other hand, however, when the network is complete, all the nodes are connected to each other, thus all the neighborhoods have equal structure, and there is no uniqueness. The neighborhoods’ uniqueness remains stable for almost all the possible values of average degree, and it starts decreasing only when the network is almost complete (see Fig. 1a for an illustration for networks with 100 nodes, and Supplementary Fig. S1 for a comparison with networks with 200 and 300 nodes). Overall, this means that, in order to understand the behaviour of the uniqueness of neighborhoods, we can focus on small average degree values as long as we observe the transition from zero uniqueness to full uniqueness.

As seen in Fig. 1a, the larger an ER network, the larger values of average degrees are needed to observe the transitions from zero uniqueness to full uniqueness. It turns out that this behavior can be understood in the case of ER networks, due to the fact that the larger the ER network is, the smaller the network density *p* for a given average degree would be. The overall network density is exactly the same as the expected neighborhood density, which means that most of the neighborhoods remain empty for low values of *p*^46^.

If, in the neighborhood of a node, there are no edges between the neighbors, that neighborhood is entirely described by the degree of the node. Further, if all the nodes have empty neighborhoods (${\mathscr {N}}_\Delta =0$) the neighborhood uniqueness ($U_{{\mathscr {N}}}$) is equal to the degree uniqueness ($U_{k}$). In fact, this is exactly what we seen in Fig. 1a: transition from empty to non-empty neighborhoods happens before the neighborhood uniqueness transitions. In addition, the degree uniqueness values remain very low, especially for larger networks. This means that, before the neighborhood uniqueness transition, the neighborhood sizes (i.e., degrees) alone are not enough to make the nodes unique. The empty neighborhoods need to contain at least small amount of edges in order for the neighborhood uniqueness transition to take place. When this behavior is combined with the fact that smaller networks have denser neighborhoods, we can understand why the uniqueness transition is driven by the network size.

In ER networks we do not only need to resort to simulating networks for particular parameter values, but we can also derive the formulas for both $\langle U_{k} \rangle$ and ${\mathscr {N}}_\Delta$. For $\langle U_{k} \rangle$ we can write down the probability that a node has degree *k* and no other node has the same degree, and sum over all possible degrees:

5 $$\begin{aligned} \langle U_{k}^{}\rangle = \sum _{k=0}^{n-1} p_k (1 - p_k)^{n-1} , \end{aligned}$$

where $p_k$ is the probability of a node to have degree *k*, according to the degree distribution of ER networks (binomial distribution):

6 $$\begin{aligned} \begin{aligned} p_k\,&= \left( {\begin{array}{c}n-1\\ k\end{array}}\right) p^{k} (1-p)^{n-1-k} \\&= \frac{(n-1)!}{k! (n-1-k)!} \bigg (\frac{\langle k \rangle }{n-1}\bigg )^{k} \bigg (1- \frac{\langle k \rangle }{n-1}\bigg )^{n-1-k} . \end{aligned} \end{aligned}$$

Equation (5) matches the simulations as seen in Fig. 1b. We can also see that the *degree uniqueness* curve (Fig. 1b,c) has a convex shape, and the maximum fraction of nodes with unique degrees is reached when the average degree is half of the maximum one ($n - 1$). We can confirm this in general by taking the first derivative of ${\langle U_{k}^{}\rangle }$, and evaluating it at $\langle k \rangle = \frac{n - 1}{2}$, with the result being equal to 0:

7 $$\begin{aligned} \frac{d \, {\langle U_{k}^{}\rangle } }{d k}\bigg |_{{\langle k \rangle }=\frac{n-1}{2}} = 0 \,. \end{aligned}$$

To compute ${\mathscr {N}}_\Delta$, we first compute the probability that the neighborhood of a node with degree *k* is non-empty:

8 $$\begin{aligned} {\mathscr {N}}_\Delta (k) = 1 - (1-p)^{\left( {\begin{array}{c}k\\ 2\end{array}}\right) } = 1 - (1-p)^{\frac{k(k-1)}{2}} \,. \end{aligned}$$

The expected fraction of non-empty neighborhoods is then given by taking the expectation over the degree distribution,

9 $$\begin{aligned} {\mathscr {N}}_\Delta = \sum _{k=0}^{n-1} {\mathscr {N}}_\Delta (k) p_k \,. \end{aligned}$$

The uniqueness of neighborhoods in finite ER networks is driven by the sparsity of the neighborhoods, which is sensitive to the network size for a given average degree. If the two nodes have the same degree they have different neighborhoods only if the subnetwork induced by the neighborhoods are not isomorphic. Since ER networks are statistically homogeneous, this is the same probability that two ER networks of the size of the neighborhood and same edge probability as the original network are not isomorphic. Sparse ER networks have small neighborhoods, which are more likely to be empty the larger the network is given a constant expected degree of the network. As real-world (social) networks often show significantly different neighborhood density from random networks^46^, using ER networks as a model for them will likely underestimate the privacy risks. This reasoning is valid also for other large random networks which are known to show a tree-like structure. Note that Eq. (5) does not make assumptions about the degree distribution, and other degree distributions, $p_k$, could be substituted to compute the degree uniqueness.

### Uniqueness maps

**Figure 2:**
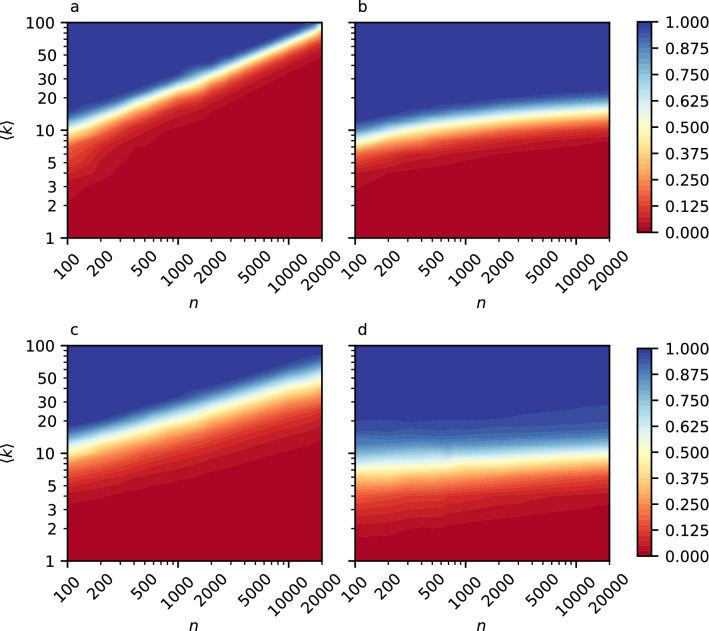
Uniqueness maps: heatmaps representing the variation of the uniqueness of neighborhoods value (in color: the blue area corresponds to a uniqueness value equal to 1, while the red area corresponds to a uniqueness value equal to 0) in networks generated according to (**a**) ER model, (**b**) WS model with probability of rewiring $\beta = 0.5$, (**c**) the BA model, and (**d**) the RGG model, as the function of the average degree $\langle k \rangle$ (on the vertical axis) and network size *n* (on the horizontal axis). The axis are in logarithmic scale. The uniqueness values are computed as the mean of 10 independently simulated networks for each average degree and network size value. Note that, even if not clearly shown in this figure, in networks with 100 nodes, the maximum possible value of average degree is 99, where $U_{{\mathscr {N}}}= 0$ (see Fig. 1 for an example of the uniqueness behaviour in ER networks with 100 nodes).

We construct *uniqueness maps*, which show the value of the uniqueness of neighborhoods as a function of both the average degree and the network size. In addition to ER networks, we include in our analysis models with neighborhood structure which is dense and independent of the network size (a property which is more typical of real-world social networks). We build *uniqueness maps* for ER, WS (with $\beta = 0.5$), BA and RGG models, with number of nodes from 100 to 20,000 and average degree from 1 to 100. The maps of the four models can be seen in Fig. 2.

We can see that the area where both anonymous nodes and uniquely identifiable nodes exists simultaneously is a small band running across each heatmap. For instance in Fig. 2b, c this region is constrained between values of average degree of $\approx 2$ and $\approx 30$. This range of values is small compared to all the possible average degrees, especially for bigger networks (e.g. a network with 1000 nodes could have average degree from 0 to 999, one with 10,000 nodes could have average degree from 0 to 9999).

That is, nodes in those networks are mostly either almost fully anonymous, or vulnerable to re-identification, and the transition between these two states happens over a relatively small range of average degree values. The area where networks are almost fully anonymous is the ones with low values of average degree, and we can see that the number of anonymous nodes increases more slowly as more local structure is present in the network. In fact, for networks such as ER (Fig. 2a) or BA (Fig. 2c), the uniqueness of neighborhoods is significantly lower compared to the other models when the number of nodes is high—with even higher values of average degree. In ER networks, at the limit of infinitely large networks and a given average degree, any neighborhood is almost surely empty and we can find two nodes that have structurally equivalent neighborhoods (i.e., belonging to the same isomorphism class). BA networks are known to be, in general, locally more clustered than ER networks, and the clustering coefficient decreases with the size. The power-law distribution could potentially make nodes diverse from each other, and has an influence on how the neighborhoods would be structured. However, this is not enough to explain the uniqueness of neighborhoods, and, in large and sparse networks, the behaviour of BA would be similar to the ones of ER, with many empty neighborhoods.

Conversely, this is not true for the WS and RGG models, which have non-empty neighborhoods independent of the network size. Further, for those networks, uniqueness is not strongly influenced by the number of nodes. Obviously, if we go to the infinite size, the number of anonymous nodes would increase, but the slope of the uniqueness’ boundary is definitely lower than in networks that are locally tree-like. Compared to the RGG, the WS model shows a faster transition area from the two extreme values of uniqueness.

The behaviour of the WS changes as $\beta$ changes, (see Supplementary Fig. S.2 for uniqueness maps of WS with $\beta$ equal to 0.25 and 0.75). If $\beta$ was equal to 1, the figure would have been similar to the one of an ER network, while, if $\beta$ was lower (e.g. at 0.25, but not close to 0), the uniqueness would be even less dependent on the network size, and the transition between the value of uniqueness from 0 to 1 would have been even faster. If $\beta$ was instead 0, then there would not be unique neighborhoods at all, as the WS graph would be in its initial regular ring lattice configuration, where all the nodes have exactly the same neighborhood structure.

The monotonicity of the uniqueness as a function of network size and average degree, and the two clear distinct areas of zero and full uniqueness that result, imply that we can describe the uniqueness in these models by simple rules. However, the two areas are not necessarily comparable in terms of the network anonymization problem, as it is likely that the further we are away from the transition point in the area of full uniqueness, the more changes we would need to make to mitigate an attack’s risk using the known anonymization algorithms (see “Privacy in social networks”). Too many of these changes make the network very different from the original one, and, consequently, useless for further analysis.

**Figure 3:**
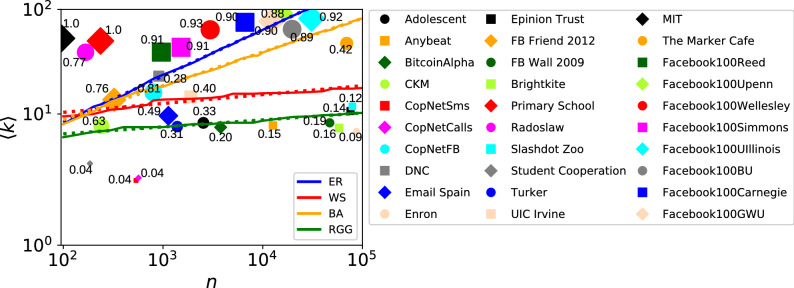
Lines representing uniqueness’ boundary ($U_{{\mathscr {N}}}{} = 0.5$) in ER (blue), WS with $\beta = 0.5$ (red), BA (orange), and RGG (green) network models, in a log-log scale, and uniqueness of neighborhoods for 30 real-world networks (reported also in Table 1). The horizontal axis represents the network size *n*, while the vertical axis represents the average degree $\langle k \rangle$. The area below the lines is the one with uniqueness $< 0.5$, while above the lines the uniqueness is $> 0.5$. The continuous lines are the ones computed with the simulations during a binary search process, while the dashed lines are the corresponding linear fits (that have equations $log(y) = m \times log(x) + c$). The dots correspond to the uniqueness of neighborhoods of the real world networks listed on the right, and their size is in according to their value of $U_{{\mathscr {N}}}\,$. The dots are placed in correspondence to the average degree and network size value of the networks.

The shape of the uniqueness maps are relatively simple: the fully unique area and non-unique area are separated by a band that appears linear and roughly constant width in the double logarithmic figures. To characterise the change of uniqueness, we focus on the functional form of the middle of this band, which is a boundary above which more than half of the neighbourhoods are uniquely identifiable and below which they are not. We use a stochastic version of the binary search algorithm, explained in Supplementary Section B, to estimate this uniqueness’ boundary, which we define as the curve corresponding to $U_{{\mathscr {N}}}= 0.5$. As Fig. 3 shows, we found that the uniqueness’ boundary has a linear trend depending on the network size and average degree. This finding implies a simple approximate laws of uniqueness for our networks models, which in turn allows us to predict if networks of given size and density are within the almost fully anonymous or almost fully vulnerable state. This prediction could serve as a proxy to understand the uniqueness of real-world networks, and to have an idea of how we can modify the network to pass from the unique area to the anonymous one. In the next section, we discuss how the uniqueness of some real-world networks relates to the ones in the analyzed models, and, based on that, illustrate an alternative strategy to mitigate the re-identification risk from neighborhoods in networks.

## Uniqueness of neighborhoods in empirical networks

In this section, we compare the theoretical results obtained through the analysis of network models, with measurements from 30 empirical social networks (with size varying from 100 to 100,000), sampled from the Index of Complex Networks (ICON)^52^. The networks we use in our analysis and the related measures are reported in Table 1. Figure 3 shows the value of $U_{{\mathscr {N}}}\,$ for those networks, compared to the uniqueness’ boundary ($U_{{\mathscr {N}}}\,$ $= 0.5$) of ER, WS, BA and RGG, estimated using binary search.

In Table 1, we also report the expected uniqueness for the four previously considered models, ER, WSS, BA and RGG, and the configuration model (CM) (computed as the mean of 10 realizations), with the same size and average degree of each of the 30 empirical networks. The configuration model generates random networks with a given degree distribution. We use the configuration model to generate networks with the same degree sequence as the real-world ones, to understand whether the specific degree distribution has influence on the uniqueness of neighborhoods. Note that for large and sparse networks, the configuration model generates networks with low clustering clustering coefficient. We use the implementation of the configuration model from^53^, avoiding to get parallel edges and self-loops (if they appear during the generation process, they will be rewired).

From Fig. 3, we can see that models generally approximate the uniqueness of neighborhoods of empirical networks well, especially the RGG, which is the model with the most pronounced local structure among the ones we analyze.

The ER model is the worst model in terms of representing the real-world uniqueness’ trend. Networks that are large and have high average degree, such as several of the *Facebook* networks, have a high uniqueness value, but they are close to the uniqueness boundary point. However, the transition between the “identifiable” state and the “anonymous” one is fast also for ER, thus it can approximately capture high values of uniqueness for smaller *Facebook* networks such as *Penn*, *Carnegie*, *Wellesley* and *Reed*. Further, the very small networks *MIT* and *Primary School* have a very high average degree compared to their size, which is also reflected in their relatively high value of uniqueness. The BA model is also generally not a good predictor of the uniqueness of neighborhoods, as it has a similar behaviour to ER. We can see that the values of uniqueness it produces are generally slightly higher than ER. This is due to the fact that nodes in BA are more heterogeneous than ER (because of the degree distribution), and the clustering coefficient is higher than ER). Moreover, the transition phase between the two extreme cases is slower, as can be seen from Fig. 2c. The WS and RGG models are in most cases better predictors of uniqueness than the ER and BA model. In general, for non-extreme values of uniqueness, the WS model underestimates the uniqueness and RGG overestimates it, so that the real value is somewhere between these two. This includes networks like *Slashdot Zoo*, *Epinion Trust*, *Enron*, *Brightkite*, *FB Wall 2009* and *Anybeat*, which are large but have a low value of $U_{{\mathscr {N}}}\,$. They are in the zone of $U_{{\mathscr {N}}}\,$ equal to zero for the ER model and WS is, in some cases, better suited for them than ER. For some of those networks (*Slashdot Zoo*, *Epinion Trust*, *Slashdot Zoo*, *Anybeat*), CM is the best predictor of uniqueness, generating values close to the original one. The heterogeneity of the degree distribution per-se, however, is not enough to explain the uniqueness of neighborhoods, as discussed earlier in “Degree and neighborhood uniqueness in Erdős–Rényi networks” and as can be seen from the values of $U_k$ in Table 1. This means that the specific degree distribution plays a role in how dense the single neighborhoods are, with the appearance of triangles to make them difference from each other. However, the clustering coefficient of the CM is lower than in the original networks (e.g. *Brightkite*’s average local clustering coefficient is 0.172, while the one of the corresponding CM is 0.005; *DNC* has average local clustering coefficient of 0.494, and the corresponding CM has 0.294), meaning that the structure is not completely reconstructed by this model. This is a known result of the CM, as the clustering gets smaller as the size grows^54^. CM predicts well the uniqueness of neighborhoods also in other networks than the ones mentioned above (e.g. *Radoslaw* or *FB friends 2012*, while in other cases more clustered models like the RGG are better. For instance, this is valid for *CKM*, *Adolescent Health Survey*, *Email Spain*, *Facebook100Wellesley*, *FB Wall 2009*. In those cases, CM produces very low values of uniqueness and, when the network is large, like in *FB Wall 2009*, this is due to its characteristic of generating graphs with low clustering.

On one hand, these results confirm the importance of the local structure in making nodes unique. On the other hand, it shows that if nodes are arranged in a specific configuration (according to the degree distribution), then local triangles would appear and contribute to the emergence of unique neighborhood structures.

There are also some outliers such as *DNC* which is a network where two users are connected if they received the same email, or *The Marker Cafe*, which is an Israeli social network, where one user is connected to another if one is part of the circle of the other. Further, in some of the networks, the degree distribution is extremely skewed, some due to sampling where only links incident to certain population are sampled or sampling of one communication channel^55^. This can explain the uniqueness (very low degree nodes are likely to be non-unique). While the four models we analyze in “Uniqueness maps” do not take into account skewness of degree distributions, the CM replicates the degree distribution of networks, being the best predictor of uniqueness in those cases.

Obviously, models are not perfect. For instance, many of them produce values of uniqueness equal to 1, even when the empirical networks have a slightly lower value. The models have indeed a very fast transition between very low value of uniqueness and almost maximum one. These extreme values may not be present in real-world networks, which have more heterogeneity than our network models. In any case, there is an overall pattern that emerges: as predicted by the WS and RGG models, if a network has average degree much higher than 10, there is going to be considerable privacy issues, and if it is much smaller than that then there are almost no privacy issues (at least in terms of uniqueness of neighborhoods) even without an anonymization procedure.

**Table 1:** 30 real-world network datasets’ basic measures (number of nodes *n*, number of edges *m*, average degree $\langle k \rangle$, average local clustering coefficient *C*), degree uniqueness $U_k$, uniqueness of neighborhoods $U_{{\mathscr {N}}}\,$, and expected $U_{{\mathscr {N}}}\,$ values for the corresponding ER, WS, BA, RGG, and CM models (with the same number of nodes and average degree), computed as a mean of 10 realizations.

***Net. name***	*n*	*m*	$\langle k \rangle$	*C*	$U_{k}$	$U_{{\mathscr {N}}}\,$	$\langle U_{{\mathscr {N}}}^{ER} \rangle$	$\langle U_{{\mathscr {N}}}^{WS} \rangle$	$\langle U_{{\mathscr {N}}}^{BA} \rangle$	$\langle U_{{\mathscr {N}}}^{RGG} \rangle$	$\langle U_{{\mathscr {N}}}^{CM} \rangle$
*Student Cooperation*^56^	185	311	3.362	0.635	0.005	0.037	0.024	**0**.**035**	0.016	0.11	0.016
*CopNetCalls (Copenhagen Network Study - Calls)*^57^	568	697	2.454	0.139	0.004	0.039	0.005	0.001	0.015	**0**.**034**	0.011
*CopNetSms (Copenhagen Network Study - Sms)*^57^	536	621	2.317	0.155	0.0	0.044	0.006	0.002	0.012	**0**.**028**	0.008
*Enron emails*^58^	87273	299220	6.857	0.119	0.002	0.090	0.0	0.001	0.002	0.151	**0**.**067**
*Slashdot Zoo*^59^	79116	467731	11.823	0.058	0.001	0.119	0.0	**0**.**138**	0.0004	0.658	**0**.**100**
*Epinion Trust*^60^	75879	405740 2	10.694	0.137	0.002	0.143	0.0	0.05	0.006	0.545	**0**.**100**
*Anybeat*^61^	12645	49132	7.770	0.203	0.005	0.148	0.0	0.032	0.01	0.323	**0**.**144**
*Brightkite*^62^	58298	214078	7.353	0.172	0.002	0.157	0.0	0.014	0.003	**0**.**204**	0.25
*FB Wall 2009 (Facebook wall posts)*^63^	46952	193494	8.242	0.107	0.0004	0.193	0.0	0.015	0.006	**0**.**304**	0.007
*BitcoinAlpha (trust network)*^56^	3783	14124	7.467	0.176	0.006	0.195	0.002	0.058	0.025	0.354	**0**.**161**
*DNC (Democratic National Committee) emails*^64^	906	10429	23.022	0.494	0.026	0.283	0.544	1.0	0.71	0.98	**0**.**442**
*Turker (Amazon Mechanical Turkers)*^65^	1389	5267	7.585	0.291	0.002	0.306	0.01	0.107	0.052	0.424	**0**.**244**
*Adolescent Health survey*^66^	2539	10455	8.235	0.146	0.0	0.329	0.004	0.071	0.055	**0**.**47**	0.008
*UIC Irvine students (Facebook like)*^67^	1899	13838	14.573	0.109	0.016	0.400	0.025	0.654	0.203	0.919	**0**.**386**
*The Marker Cafe*^68^	69413	1644849	47.393	0.186	0.005	0.424	0.002	1.0	0.185	0.988	0.387
*Email Spain (Universitat Rovira i Virgili)*^69^	1133	5451	9.622	0.220	0.006	0.492	0.02	0.292	0.100	**0**.**659**	0.204
*CKM physician social network*^70^	241	924	7.668	0.311	0.016	0.634	0.11	0.295	0.137	**0**.**528**	0.199
*FB friends 2012 (Facebook friendships)*^71^	324	2218	13.691	0.465	0.03	0.762	0.422	0.952	0.45	0.905	**0**.**614**
*Radoslaw (Manufacturing company emails)*^56^	167	3251	38.934	0.591	0.155	0.766	1.0	1.0	1.0	1.0	**0**.**766**
*CopNetFB (Copenhagen Network Study - Facebook)*^57^	800	6429	16.072	0.315	0.017	0.81	0.175	**0**.**945**	0.465	0.948	0.51
*Facebook100UPenn*^72^	14916	686501	92.048	0.213	0.004	0.882	**0**.**926**	1.0	0.998	1.0	0.763
*Facebook100BU*^72^	19700	637528	64.723	0.190	0.003	0.886	0.107	1.0	**0**.**797**	0.998	0.613
*Facebook100Carnegie*^72^	6637	249967	75.325	0.278	0.011	0.896	**0**.**997**	1.0	0.999	1.0	0.044
*Facebook100GWU*^72^	12193	469528	77.015	0.217	0.006	0.903	0.747	1.0	**0**.**999**	**0**.**999**	0.767
*Facebook100Reed*^72^	963	18812	39.069	0.318	0.03	0.906	1.0	1.0	1.0	0.997	**0**.**866**
*Facebook100Simmons*^72^	1518	32988	43.462	0.314	0.023	0.907	0.999	1.0	1.0	0.998	**0**.**859**
*Facebook100UIllinois*^72^	30809	1264828	82.081	0.214	0.002	0.920	0.127	1.0	0.89	**0**.**999**	0.642
*Facebook100Wellesley*^72^	2970	94899	63.905	0.264	0.017	0.932	1.0	1.0	1.0	**0**.**999**	0.066
*MIT*^73^	96	2539	52.895	0.751	0.302	1.0	**1**.**0**	**1**.**0**	**1**.**0**	**1**.**0**	**1**.**0**
*Primary School dynamic contacts*^74^	236	5899	49.991	0.501	0.009	1.0	**1**.**0**	**1**.**0**	0.999	**1**.**0**	**1**.**0**

The table is sorted by increasing value of $U_{{\mathscr {N}}}\,$. The models’ expected $U_{{\mathscr {N}}}\,$ values that are closer to each real network are highlighted in bold (if two models produced the same value, they are both highlighted).

## Mitigating neighborhood attacks

In the previous section, we have seen that, in terms of understanding privacy, models can be a good approximation for real-world social network data, and that, typically, social networks are better approximated by models with a clustered local structure, such as the RGG. Ultimately, we are interested in sharing network data while protecting user’s privacy or, at least, mitigating the risk of identity disclosure, while keeping the data useful for exploration and analysis. Due to the presence of two distinct areas with the almost maximum and minimum values of $U_{{\mathscr {N}}}\,$, we can first, due to the models, form an idea of “how far” a network is from the area where all the nodes are anonymous ($U_{{\mathscr {N}}}\approx 0$). This knowledge can, depending on the anonymization strategy, also help in understanding how many modifications we need to make the network anonymous. We can perform these estimations even before we have the full data available just by having an estimate on the network size and density.

Inspired by the strong relationship between average degree and uniqueness, we now investigate a simple strategy to mitigate neighborhood attack based on lowering the average degree by random edge sampling. At first this trivial method might seem inferior to the methods that try to minimize the number of changes to the network. However, there are two main advantages of sampling: first, sampling is extremely simple compared to the optimisation heuristics, making it practical and even possible to use already at the data collection phase, even before collecting the whole network (which would be impossible with the current optimisation methods); second, random sampling produces statistically tractable changes to the network—as opposed to optimisation algorithms for which one in practise needs to assume that the changes are in the worst possible places. For this reason, random edge sampling as an effort to lower the de-anonymization risk cannot be directly compared to other methods described in “Privacy in social networks”, which mostly make changes to the networks by adding edges arbitrary positions in the network. These methods are typically mostly evaluated with the difference of certain specific measures before and after anonymization, while while we, instead, try to reconstruct original measures with statistical methods. In addition to serving as a simple anonymization method, random edge sampling confirms our previous results for random networks that lower average degrees lead to lower uniqueness in real data.

As we discuss below, the application of this method need to be coupled with analysis of also other risk factors prior to data sharing (e.g. the presence of additional indicators or other public datasets which could lead the re-identification of a node by other means), but the method is simple, intuitive and practical, as it could potentially be applied already in the data collection phase.

### Random sampling of edges

By exploiting the fact that the uniqueness in real-world social networks is heavily influenced by the average degree, we investigate an approach to mitigate neighborhood attack in social networks with respect to uniqueness of neighborhoods by randomly sampling edges. Here the idea is that we uniformly randomly choose a set of edges in the data to keep and remove the rest. This approach leaves the number of nodes unchanged, while lowering the average degree and, consequently, lowering the uniqueness of neighborhoods. Random edge sampling makes more statistical guarantees on the final analysis, allowing the estimation of original measures through statistical methods^75^. Conversely, with existing optimization-based methods, reversing the effect of the anonymization algorithm could be difficult.

The disadvantages of random edge sampling is that each piece of information that is shared is actually true. That means, each published edge actually exists, revealing real relationships between the entities represented. In addition, a careful analysis of risk deriving from the presence of possible nodes’ attributes and node identities would need to be carried out, in order to decide the best anonymization or pseudo-anonymization method. However, if the nodes have no attributes or no unique-identifier attributes, the proposed approach could be suitable. In the presence of attributes, in any case, the computation of uniqueness should be carried out taking into account those as well. In this paper, we focus only on structural uniqueness, without considering any nodes’ attribute.

**Figure 4:**
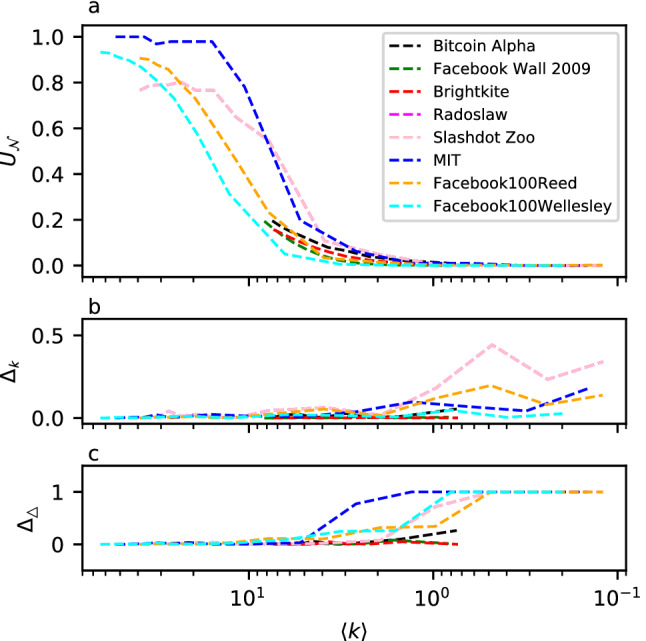
Results for 8 networks listed in Table 1 after uniform random edge-sampling, i.e., when a fraction of randomly chosen edges are removed. The horizontal axis indicates the average degree $\langle k \rangle$ of the networks after sampling (removal of random edges). The sampling rate is varied between no links being removed (where the average degree is that of the original network) and a rate which yields a low enough average degree such that we reach zero uniqueness ($U_{{\mathscr {N}}}{} = 0$). The curves start from the average degree value of the original network. (**a**) Effect of uniform edge sampling on the value of $U_{{\mathscr {N}}}\,$(on the vertical axis): as we sample edges, the values of the average degree $\langle k \rangle$ gets lower as well; (**b**) error of the estimation of the degree of the nodes (average over all the nodes in the network) in the networks with Eq. (10); (**c**) error of the estimation of the amount of triangles in the networks with Eq. (11). The error in these estimates is the difference, in absolute value, between the original value and the estimated one.

Figure 4a shows the effect of the random sampling of edges on the average degree and uniqueness of neighborhoods. In general, the higher the amount of sampling we perform, the lower the numerical value of those two measures would be. We report results for 8 of the networks we listed in Table 1. We chose those networks to have different size, average degree, and value of uniqueness.

We can see in the figure that the real-world networks follow the same pattern we observed for our model networks in “Uniqueness maps”: the uniqueness monotonically decreases as the average degree decreases. Further, there is a transition-like behavior close to the uniqueness boundary, such that networks that have a high original value of uniqueness ($\approx 1.0$) first experience a slow decrease after the initial samplings and, then, a very rapid fall in the uniqueness values, until they get more stable again towards the lowest values of uniqueness (i.e., with the highest sampling rates). In fact, it seems that, when sampled, networks display a rapid transition (represented by the rapid decreasing of the uniqueness’ value) between a state where nodes are almost all unique and a state where there is no unique node. In models, the area in between the two extreme zones was also relatively small, meaning that the range of average degree needed to pass from the “identifiable” state to the “anonymous” state is relatively narrow.

As we sample edges, we are modifying the neighborhoods at random, not necessarily targeting the unique neighborhoods but all of them uniformly. In a large network this could mean that few unique neighborhoods would persist for very high edge-removal rates. For this reason, in real-world applications, we would not necessarily want to sample until we reach zero values of uniqueness, as this could require us sample extensively compared to reaching the state of having uniqueness close to zero. In fact, even though few neighborhoods could still be unique at this stage, they would nevertheless be modified because of the effect of sampling on their edges. Stated differently, there would be cases where a neighborhood is unique, but modified to such extent that we could find at least one other sampled neighborhood which could equally likely have been the original neighborhood. To be more precise on this point, we would need to perform a further study on “how far” the neighborhoods in the network are from the original one and from other neighborhoods. This analysis would require measuring their edit-distance and the actual risk of a neighborhood to be re-identified. Such an analysis is, however, beyond the scope of our study.

### Error evaluation and correction

As we are sampling edges uniformly, we can reconstruct measures of the original network, given the sampling rate that can be provided together with the sampled data.

Note that the ability to evaluate and correct the error is specific to this method, something which is not trivially possible to do with existing methods. For this reason, edge sampling is not directly comparable with existing algorithms. The performance of anonymization algorithms in the literature is normally measured by the number of changes (e.g. edge additions or removals) or by the difference in key network statistics after the anonymization procedure. The sampling approach we take here clearly leads to more changes and larger differences in the network statistics. However, there is no way to correct for the biases in the optimization-based anonymization methods. It is also difficult to derive confidence intervals for the various network statistics for these methods, and to do so one would likely need to assume that the changes are in the places that change these network statistics the most. This cautious assumption would probably render most of the anonymized networks unusable for various purposes.

We show that data anonymized via edge sampling allows the end user to make unbiased estimates and related error estimation of network statistics by employing network sampling theory^75^. As an example of this approach, in Fig. 4b, c we show the error estimation of two basic network measures, degree of the nodes, and number of triangles, derived from the sampling of edges in the 8 networks reported in Fig. 4a.

Each node *i*’s degree $k_{i}$, after sampling, can be estimated with the following formula:

10 $$\begin{aligned} \widehat{k}_{i} = \frac{k_{i}^{s}}{s} \,, \end{aligned}$$

where *s* is the sampling rate and $k_{i}^{s}$ is the degree of node *i* observed in the network after sampling. The amount of triangles $\bigtriangleup$ in the network can be estimated with:

11 $$\begin{aligned} {\widehat{\bigtriangleup }} = \frac{\bigtriangleup ^{s}}{s^{3}}\,, \end{aligned}$$

where $\bigtriangleup ^{s}$ is the amount of triangles observed in the network after sampling. The error in these estimates is the difference, in absolute value, between the original value and the estimated value. For each sampling rate and each network considered, we show the error of the estimation in Fig. 4b,c. In terms of degree error, we show the average error over all nodes. We see that the error is relatively small for even relatively high values of sampling (and, consequently, low values of average degree), especially for the degree. The error is a bit higher (slightly above 0.2), for the triangle-count. The latter is a slightly more complicated measure than the degree, so it is expected that, with higher sampling rate, we obtain an higher estimation error. The higher the sampling rate, the less the possibility to re-identify nodes as well. This can also be seen as a privacy-utility trade-off. However, as we mentioned before in “Random sampling of edges”, we could not necessarily need to sample until we reach the minimum uniqueness. In any case, the average errors on these networks are still quite low, confirming that our method can give some statistical guarantees on any measure, allowing at least a certain confidence in estimating statistics from the original network.

## Discussion

In this paper we studied the uniqueness of neighborhoods in networks, discovering that there are regularities in the uniqueness behaviour of network models, by investigating its dependencies on average degree and network size. We have seen that, in models, there is a narrow boundary area which separates two zones where nodes are either almost fully vulnerable to identification ($U_{{\mathscr {N}}}\approx 1.0$) or almost fully anonymous ($U_{{\mathscr {N}}}\approx 0$). We find that the middle-point of this are depends linearly on the networks size, but in models that produce locally dense structures, the uniqueness depends only slightly on the network size.

Inspired by this finding, we have shown that randomly sampling edges can be an effective approach to lower the identity disclosure risk. This approach provides statistical guarantees when analyzing the shared data, which is an advantage compared to the optimization-based anonymization algorithms, which are optimized for specific purposes and where the effect of the anonymization on the network statistics could be hard to understand and reverse.

We have shown that, often, models with dense local structure such as the RGG predict the real values of the uniqueness relatively well. In other cases, the degree distribution plays a role in how the links are arranged in the network and, consequently, how dense a network is. Consequently, the configuration model can be a good predictor of the uniqueness of neighborhoods, despite the degree alone not explaining the number of unique neighborhoods. The configuration model produces networks with lower clustering than the original ones, meaning that even fewer edges in the local neighborhoods, in combination with the degree distribution, can contribute to make a node unique.

A number of questions still remains open. For example, we could also study what happens in the “unique” zone of a network, if the average degree keeps growing. As the networks move further away from the uniqueness’ boundary, and the uniqueness reaches its maximum value, we do not know whether the edit distance between the neighborhoods increases or remains stable. Even though we would expect it to increase, a further study is needed to confirm. This understanding would be useful for more accurately estimating if a network is “too far” from the anonymous area, meaning that lowering the uniqueness of neighborhoods via any anonymization process would lower the utility in a radical way, making the final analysis much less useful.

Our edge-sampling method introduces uncertainty even before the resulting uniqueness of the full network is zero, as it modifies the neighborhoods anyway. It could therefore be possible to sample less edges. The current algorithms run until they reach uniqueness equal to zero, introducing probably more modifications than needed. To better understand when to stop introducing changes, we could study the best guess of an attacker (for instance by computing the edit distance between the target neighborhood and the existing ones) in re-identifying a certain target node after the network has been anonymized with existing methods or sampled with uniform edge-sampling.

The simplicity of the random edge sampling could make it useful in some use cases. For example, one could implement it in advance before data is collected, for example by designing the data collection in a way that only a sample of links are collected. This could be especially useful in studies where there is a cost associated with collection of each link. Clearly edge sampling also comes with obvious limitations. For example, links are still fully preserved (revealing actual relationships) and only true information from the original network is shown. Moreover, our uniqueness’ study is carried out on networks without any node or edge attributes. This types of networks are of interests when studying social networks from a structural point of view, where a nodes’ neighborhood could be sensitive from a privacy point of view for revealing, for instance, the presence of additional relationships or nodes’ attribute which should be kept private. However, if nodal feature are present, those may also be taken into account in the computation of uniqueness, as they could act as identifiers, and could potentially help the attacker to identify targets.

The current trend of research for privacy-preserving data sharing is moving towards synthetic data generation, for example applying generative models with differential privacy^27,28^. This line of methods have some clear advantages over sharing modified networks. Differential privacy can guarantee certain amount of privacy against any kind of attack scenario, whereas we only studied neighborhood attack. One could also have studied node automorphisms or other stricter notions of privacy, but meeting these guarantees for large networks can be practically impossible. On the other hand, synthetic data generation is only as useful as the model behind it for a specific research question. For example, stochastic block models are unable to capture many important aspects of local structure and *dK*-series do not adequately model information on the mesoscopic structures. In general, using model generated data limits its usability to questions that can be answered by analysing the model. Data anonymization is a compromise between utility and anonymity^29,76^.

As we have seen that the uniqueness of neighborhoods is affected by the network configuration, further studies can be conducted on different types of networks, such as multiplex networks, to understand how the uniqueness is affected when the attacker has multiple data sources at its disposal. One can further analyse additional and more complicated models, that take into consideration specific nature of the network data and processes generating it. Moreover, based on our findings, models that retain the degree distribution while having a controlled density could be taken into consideration in additional studies^77^.

In general, networks are difficult to anonymize and neighborhoods are certainly not the only aspect to take into account when considering the privacy risk in data sharing. However, structural privacy is an additional point that should be certainly considered, and neighborhoods may be a crucial threat for identifying a certain entity, especially if the attacker has domain-knowledge and sensitive attributes are shared with the nodes (even if nodes’ identity are dropped).

Random network models are in general useful for two purposes: for theoretical understanding of the underlying phenomena and as proxies for real networks. For example, spreading processes on networks are routinely and successfully analysed using random network models. Here we have explored these two purposes in the context of network anonymization. Our findings bring theoretical understanding to the factors making some networks vulnerable to neighborhood attack and others not. Further, they allow one to estimate the risk even without seeing the network data. This aspect can be useful for example before data collection, to justify why a network dataset is sensitive without sharing it, or when deciding if it is even worth to apply network anonymization to it.

## Supplementary Information

Supplementary Information.

## Data Availability

The datasets analyzed during the current study were previously published by other authors and are publicly available. The data can be downloaded from the corresponding repositories mentioned in the references in Table 1.
