# Supplementary material for: Privacy and uniqueness of neighborhoods in social networks
Source: Sci Rep. 2021 Oct 11;11:20104. doi: 10.1038/s41598-021-94283-5 (PMC8505500; doi:10.1038/s41598-021-94283-5)
Supplement: Supplementary file 1 — Supplementary Information. [file 41598_2021_94283_MOESM1_ESM.pdf]

Supplementary Information

A Additional figures

In this appendix, we show additional plots regarding the uniqueness of neighborhoods and degree uniqueness in network models, in order to let the reader have a more complete picture of the behaviour of such models.

Fig. S.1 shows the uniqueness curve for the complete range of average degree (from 0 to $n-1$) for ER networks of size 100, 200, 300. The values of uniqueness are computed as a mean over 10 realization of each ER configuration. We can see that the value of uniqueness goes to one (maximum value) almost immediately for all the three networks, and it experiences a dramatic fall only towards the real end, when the network is almost complete (that is, every node is connected to any other).

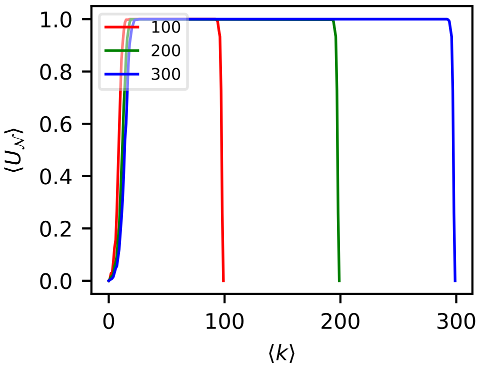

Figure S.1. Expected uniqueness of neighborhoods $\langle U_{\mathcal{N}} \rangle$ (in a linear scale), in ER networks of size 100, 200, 300 (computed as the mean of 10 independent network realizations), for the complete range of average degree $\langle k \rangle$ (from 0 to $n-1$).

Fig. S.2 shows the uniqueness maps for WS networks with probability of rewiring β of 0.25 ((a) and 0.75 (b). The higher the probability of rewiring, the closer the WS network would be to a random network, like the ER. In fact, we can see that the map of WS with $\beta = 0.75$ is close to the map of the ER network in Fig. 2a. It would be almost the same at the extreme, with $\beta = 1$ (i.e. all the edges are rewired, realizing, in practice, random network). As we observed that the uniqueness' boundary has a linear trend, we can see that this similarity is reflected by the slope of the $\langle U_{\mathcal{N}} \rangle = 0.5$ curve. On the other hand, when $\beta = 0.25$, the network is much more dense, consequently the uniqueness' behaviour is more similar to the RGG model, shown in Fig. 2c. However, if $\beta = 0$, the network would be a regular lattice (as per the initial configuration of the WS model), thus the neighborhoods of all nodes would be isomorphic to each other, and the network would have a uniqueness of neighborhoods' value equal to zero. The slope of the WS network with $\beta = 0.5$ (Fig 2b) is, as expected, in between the ones with β to 0.25 and 0.75.

B Stochastic binary search algorithm

In this appendix, we explain the modification to the binary search algorithm we use in subsection 4.2. We run the binary search to estimate the uniqueness boundary ($U_{\mathcal{N}} = 0.5$) in network models and better compare $U_{\mathcal{N}}$ in different models. The algorithm looks for a certain value of uniqueness in a network generated according to a model with a given number of nodes, in a range of average degree values delimited by two extremes.

Our algorithm is a stochastic and continuous version of the classic binary search. The algorithm searches for a target value (in our case, 0.5) in a certain range, evaluating first the extreme values of the interval and, if those are not the ones we are looking for, evaluating the middle value. If the middle value corresponds to the target value, the algorithm stops, otherwise it continues the search process recursively, until it finds the target value. The interval used in the new evaluation corresponds to either the lower part of the interval (delimited by the original lower extreme and the middle value), or the upper part (delimited by the middle value and the original upper extreme). The algorithm stops also if the extremes of the interval we are evaluating are too close to each other.

In our case, to decide on which side of the interval to move, we exploit the fact that, with a fixed network size, we know from the simulations that the uniqueness grows with the average degree (at least in the sparse region). Consequently, if a

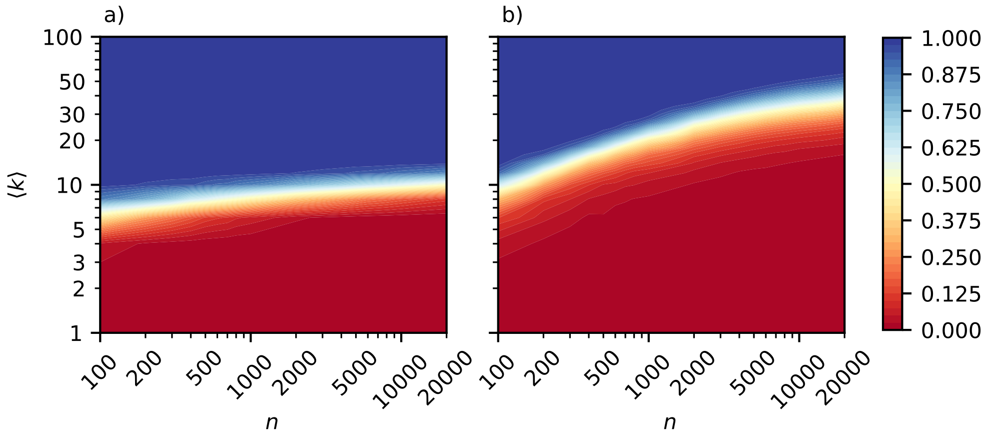

Figure S.2. Uniqueness maps of WS networks with probability of rewiring $\beta = 0.25$ (a) and $\beta = 0.75$ (b). The heatmaps represent the variation of the uniqueness of neighborhoods value (in color: the blue area corresponds to a uniqueness value equal to 1, while the red area corresponds to a uniqueness value equal to 0), in function of the average degree $\langle k \rangle$ (on the vertical axis) and network size n (on the horizontal axis). The axis are in logarithmic scale. The uniqueness values are computed as the mean of 10 independently simulated networks for each average degree and network size value. Note that, even if not clearly shown in this figure, in networks with 100 nodes, the maximum possible value of average degree is 99, where $U_{\mathscr{N}} = 0$ (see Fig. 1 or Fig. S.1 for an example of the uniqueness behaviour in ER networks with 100 nodes).

certain average degree value we are evaluating gives a uniqueness value higher than the one we want, we move to the left (or lower) part of the interval, which is the one containing lower values; otherwise we move to the right (or higher) part, by always computing the middle value of the new interval.

To compute the uniqueness value corresponding to each average degree, we generate five networks with that average degree and the given network size, and we take the mean of the corresponding uniqueness values. Since we want certain guarantees every time we decide which new interval to evaluate (and also when to stop), we compute a confidence interval (at 99% confidence level) of the mean of the uniqueness value of the networks generated with certain parameters. We then check whether the target uniqueness value is contained in that interval: if it is not, we move either to the right or the left side; if it is, we run new simulations to have a better estimation of the real mean. If the target uniqueness value is still in the interval after a maximum number of simulation (we chose 30), then we can be confident that the evaluated average degree is the one we are looking for. Otherwise, we continue with the search. We also set a 0.02 tolerance level to the target uniqueness value. That means, if we find an average degree corresponding to either 0.52 or 0.48, the search process is considered successfully ended.
